# Resolution of Sonographic Appendicitis in Pediatrics: a Point of Care Ultrasound Case-Series 

**DOI:** 10.24908/pocus.v9i1.16860

**Published:** 2024-04-22

**Authors:** Eric Scheier, Benjamin Taragin

**Affiliations:** 1 Pediatric Emergency, Kaplan Medical Center and Faculty of Medicine, Hebrew university of Jerusalem Israel; 2 Associate Director, Medical School for International Health, Ben Gurion University Sherbrooke, QC Canada

**Keywords:** POCUS, Ultrasound, Appendicitis, Pediatric

## Abstract

Studies of pediatric appendicitis treated conservatively show a considerable rate of recurrence. Point of care ultrasound (POCUS) imaging at our facility is routinely performed for abdominal pain and may be more likely than radiology-performed ultrasound to encounter cases that then self-resolve. We present a case series collected from a POCUS quality assurance review from 2019 through 2022. Five children were identified with sonographic appendicitis on review of stored POCUS images, and subsequent improvement of pain. A pediatric radiologist reviewed blinded images and agreed with the POCUS interpretation in all five cases. No child in this series received antibiotics. The national patient database was used to ensure that the patients in this series did not present elsewhere with appendicitis. We suggest that these cases represent early appendicitis that self-resolved. Patients should be aware that POCUS showed signs of appendicitis, and should seek medical attention for recurrence of symptoms.

## Introduction

Appendicitis is the single most common surgical diagnosis in the pediatric emergency department (PED) 1. Pediatric appendicitis treated conservatively (without surgery but with antibiotics) have a one-year recurrence rate of 18.6% and a five-year recurrence rate of 23.3% 2. Point of care ultrasound (POCUS) is becoming more widely accepted as a means to diagnose pediatric appendicitis. POCUS is without radiation and can be performed comfortably with adequate analgesia. Here, we present a case series of sonographic appendicitis on POCUS in children discharged without medical or surgical treatment for appendicitis. We suggest that these cases represent early appendicitis that self-resolved. This series brings awareness to pediatric emergency physicians that very early appendicitis may self-resolve, and to patients that may be at increased risk of recurrence. 

## Methods

Our pediatric emergency department (PED) sees 27,000 children (to the age of 18 years) annually. In the context of a wider study (approved by our institutional review board as KMC 0202-23), we found 414 cases of appendicitis confirmed by pathology or computed tomography (CT) from 2019 through the end of 2022, of which 171 were confirmed with POCUS. POCUS was performed on a Zonare Z.One ultrasound by ten pediatric emergency fellows and attendings with 1-6 years of POCUS experience. We use a linear, high frequency probe to diagnose appendicitis. The examination is considered positive if it demonstrates a tubular, noncompressible, aperistaltic structure in the right lower quadrant, greater than 6 millimeters in diameter, with or without secondary findings such as wall thickness above 2 millimeters free fluid, or peri-appendiceal inflamed fat 3.

Our surgical department has traditionally hospitalized children for observation if they have a negative ultrasound in the PED but a clinical examination or laboratory evaluation that cannot exclude appendicitis, or a positive ultrasound with an unremarkable laboratory and physical examination. Children without a diagnosis of appendicitis and hospitalized for observation do not receive antibiotic coverage in the PED or in the inpatient unit, but are re-examined by a pediatric surgeon. 

During the study period, five children were identified on quality assurance review to have POCUS images consistent with appendicitis, but were discharged from the PED or hospital without antibiotic therapy or surgery. The country-wide OFEK electronic medical record database, which includes all outpatient visits as well as all hospitalizations, was interrogated to verify that the children in our series did not return to a medical facility with appendicitis within at least a year from PED presentation. An experienced pediatric radiologist blinded to the clinical data reviewed anonymized images for this study and agreed with the POCUS interpretations. Scoring systems to assess the likelihood of appendicitis are not used at our facility.

## Case Reports

### Case 1

**Figure 1:**
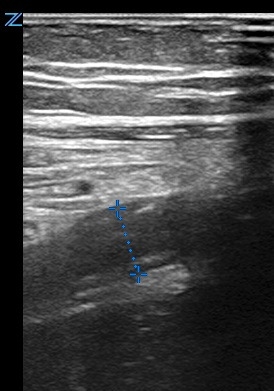
7-millimeter noncompressible appendix in long-axis.

**Figure 2:**
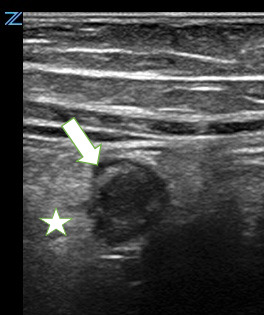
Appendix, noncompressible, inshort-axis, surrounded by echogenic mesenteric edema/ inflammation. The arrow’s tip sits on a small amount of free fluid surrounding the tubular appendix. The star sits in the center of a cloud of echogenic inflamed fat.

A 7-year-old female presented with several hours of abdominal pain and emesis, without fever or diarrhea. Visual Analog Scale (VAS) was not recorded. She had right lower quadrant tenderness, without peritoneal findings. White blood cell (WBC) count was 11.8 K/micron, and neutrophil count 10.3 K/micron. POCUS was read by the pediatric emergency fellow as unremarkable, but was flagged on quality assurance review as showing a 7-millimeter appendix surrounded by inflamed fat (Figures 1 & 2). She was discharged from the PED.

### Case 2

**Figure 3:**
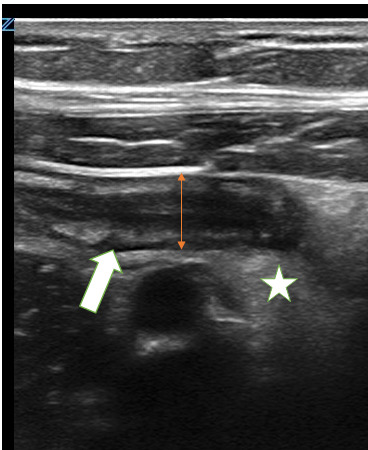
7.5-millimeter noncompressible appendix in long-axis with wall thickening and surrounding mesenteric edema/ inflammation. The arrow’s tip sits on then irregular thickened wall of appendix. The arrow sits in the center of a cloud of echogenic inflamed fat. The double arrow indicates the appendiceal diameter.

A 13-year-old female presented with hours of periumbilical pain without fever vomiting or diarrhea; VAS was 2. Examination showed bilateral lower quadrant tenderness without peritoneal signs. Complete blood count (CBC) was normal, and C-reactive protein (CRP) was 4.6 mg/dL. POCUS found an enlarged non-compressible appendix that appeared thick-walled and was surrounded by inflamed fat (Figure 3). Radiology-performed ultrasound (RADUS) found a 7.5 millimeter noncompressible tubular structure in the right lower quadrant consistent with appendicitis. She was admitted to surgery for observation. She was discharged the following morning with a nontender abdomen.

### Case 3

**Figure 4:**
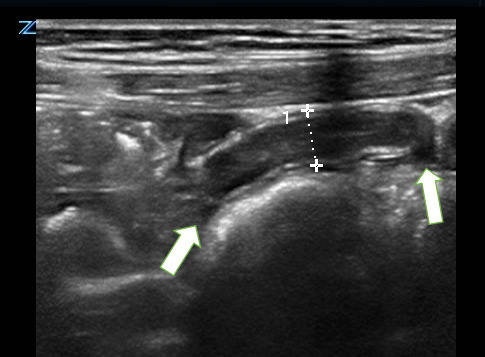
7-millimeter noncompressible appendix with wall thickening with free fluid adjacent to the tip.

A 7-year-old male presented with several hours of severe lower abdominal pain, worse with walking or bending and worse on urination. He had one episode of diarrhea, no fever, and no emesis. Past medical history is significant for a hospital admission for abdominal pain two years prior, and received a dose of intravenous antibiotics. RADUS at that time describes mesenteric lymphadenitis and an appendix thoroughly visualized with a maximum diameter of 8 millimeters and no signs of inflammation. VAS at the current visit was 5. Examination showed lower abdominal pain with guarding. CBC and CRP were unremarkable. POCUS showed a 7.3 millimeter noncompressible appendix with free fluid at the tip (Figure 4). RADUS was not completed due to noncompliance with exam. The patient was admitted to surgery overnight for observation and preparations were made to send him for abdominal CT. Examination by the attending surgeon after arrival in the surgical unit showed a soft abdomen that with mild tenderness to deep palpation. Examination 39 hours after arrival in the PED showed a soft nontender abdomen, and he was discharged on hospital day 2. 

### Case 4

**Figure 5:**
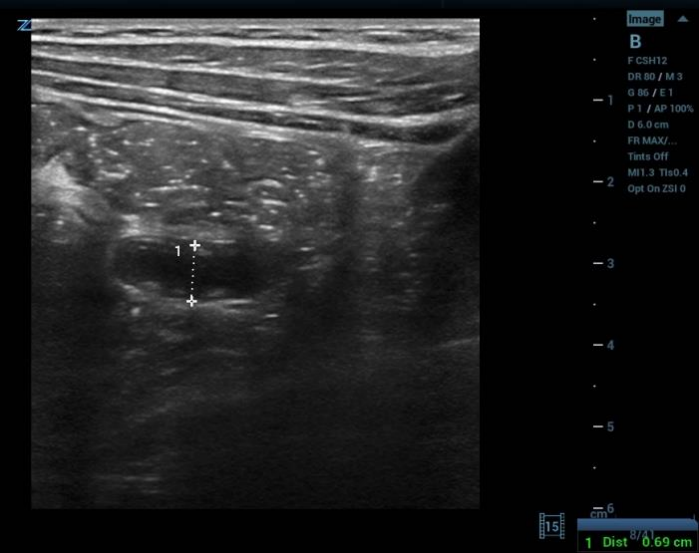
Fluid filled 6.9-millimeter appendix in long-axis, with wall thickening and surrounding mesenteric edema/ inflammation.

**Figure 6:**
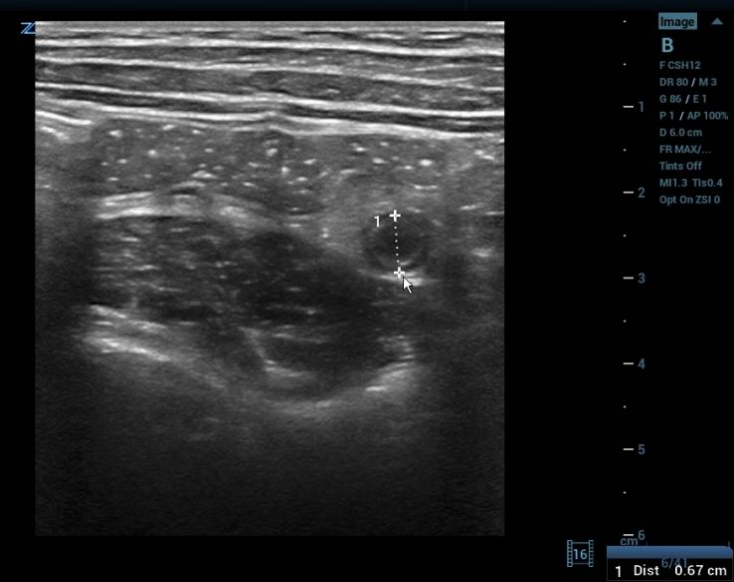
Fluid filled 6.7-millimeter appendix in short-axis, surrounding mesenteric edema/ inflammation.

A 10-year-old female presented with hours of severe abdominal pain. She reported no fever, vomiting or diarrhea; VAS was 8. CBC was unremarkable, and CRP was 3.63 mg/dL. POCUS showed a 6.9-millimeter noncompressible appendix (Figures 5 & 6). RADUS showed a 7.5-millimeter appendix with wall thickening, enlarged mesenteric lymph nodes, and a small amount of fluid in the right gutter. She was admitted to surgery, with admission orders to begin fasting and intravenous fluid in preparation for surgery. On admission, the attending surgeon described a soft abdomen with moderate tenderness to deep palpation and no peritoneal signs. On hospital day 1 she reported improvement in abdominal pain and began to eat. Repeat CBC was unremarkable, and CRP was stable at 3.56 mg/dL. Repeat RADUS again showed a 7-millimeter appendix with surrounding mesenteric inflammatory change and an appendicolith at the base, thickening of surrounding small bowel wall, and enlarged nodes, consistent with acute appendicitis. On hospital day 2, CBC was again unremarkable and CRP declined to 1.44 mg/dL. RADUS showed no change from prior. On hospital day 3 she was discharged with mild right lower quadrant (RLQ) tenderness. She returned to the PED 2 days after discharge with abdominal pain, and only mild tenderness on exam. POCUS showed a normal appendix. After surgical consultation, she was discharged.

### Case 5

**Figure 7:**
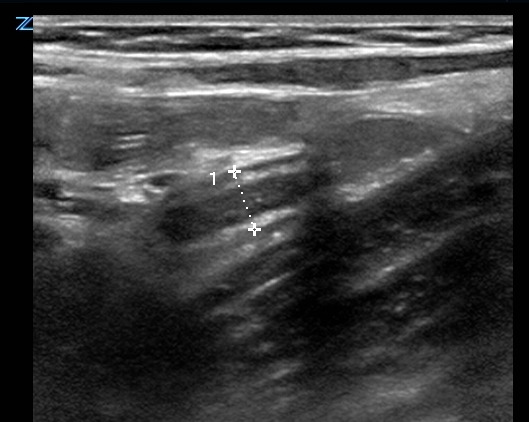
7-millimeter appendix with wall thickening in long-axis.

A 9-year-old male with a history of Celiac disease presented with hours of abdominal pain that migrated to the RLQ. He denied fever, vomiting and diarrhea. VAS was 4. Examination was significant for right upper and lower abdominal tenderness with positive Rovsing and obturator signs. CBC was unremarkable, CRP 2.37 mg/dL. POCUS showed enlarged mesenteric lymph nodes in the right lower quadrant of the abdomen, with a 7-millimeter noncompressible appendix (Figure 7, Video S1). He was admitted for observation. On hospital day 1 CBC was again unremarkable, CRP 2.27 mg/dL. RADUS found a 6-millimeter appendix partially compressible with surrounding inflamed fat, “possibly consistent with early appendicitis.” Repeat RADUS on hospital day 2 was unchanged. On both hospital days, examination by the attending gastroenterologist showed a soft abdomen with mild right lower quadrant tenderness. Examination by the attending surgeon was similar and deemed inconsistent with acute appendicitis, and the patient was discharged home. 

## Discussion

This is the first series of pediatric sonographic appendicitis that resolved without antibiotic or surgical therapy. Our series comes to bring greater awareness of spontaneous resolution of pediatric appendicitis in the context of increasing availability of POCUS in the PED.

Emergency physician performed POCUS has been shown to have a learning curve outside the context of fellowship training 4. POCUS for appendicitis in a mixed population has a pooled sensitivity 0.81 and pooled specificity of 0.87, noninferior to RADUS 5, 6. Others have found lower numbers in physicians with basic training in POCUS 3, 7.

In the context of a negative ultrasound, prior work has shown a negative predictive value of 96% when the WBC is less than 11,000 cells/micron 8. There, 25% of children with appendicitis and a normal laboratory evaluation were detected by POCUS and 75% were detected by RADUS. Thus, early in the course, laboratory evaluation may be unremarkable, and POCUS can work side by side with RADUS to prevent missed appendicitis.

Non-operative management – treatment of appendicitis with antibiotics in lieu of surgery – is becoming more frequent and, in the short-term, has a high rate of success. A systematic review of seven studies of pediatric uncomplicated (unruptured) appendicitis showed a success rate to hospital discharge of 91%^9^ and 41.7% of parents would choose non-operative management if their child had appendicitis 10. However, initiation of antibiotic therapy commits the child to hospitalization and to completing a course of intravenous and oral antibiotics.

Since the 1990s, several authors have reported on cases of spontaneous resolution of appendicitis 11, 12, 13. Cobben, et al. reported on 60 adults with spontaneous resolution of appendicitis. They showed a recurrence rate of 38%, with higher rate of recurrence in cases with an appendiceal diameter above 8 millimeters 14. Lastunen et al. studied 184 adult patients with an Adult Appendicitis Score of 11-15and less than 24 hours of symptoms. These patients were allocated to either early imaging (ultrasound and/or CT) or observation arms. Those in the observation arm were reassessed after 6-8 hours. They showed that those imaged early were diagnosed more frequently with appendicitis (72% versus 57% in the observation group) 15. This result may suggest that there are adult patients with early appendicitis who experience spontaneous resolution. Similarly, a meta-analysis of over 100,000 adult patients found a decrease in the proportion of complicated appendicitis compared with uncomplicated appendicitis during the COVID-19 pandemic year. The decrease in proportion of uncomplicated appendicitis without an increase in the absolute number of complicated appendicitis between the two time periods suggests that a certain amount of uncomplicated appendicitis resolved spontaneously during a period of limited access to health care 16. A 2007 review by the same author concluded that “spontaneous resolution of untreated, non-perforated appendicitis is common” 17.

Park et al. randomized 245 adult patients with uncomplicated appendicitis (defined as appendiceal diameter no larger than 11-millimetersand without any signs of perforation) to antibiotic or supportive therapy. The percentages of treatment failure were similar between the two groups (23.4% in the no-antibiotic group and 20.7%in the antibiotic group), further supporting the notion that spontaneous resolution of uncomplicated appendicitis may not be a rare occurrence 18.

A series of 182 pediatric patients with low-grade sonographic appendicitis (defined there as “an appendix with a smooth submucosal layer or irregular submucosal layer with increased blood flow and no appendiceal mass, abscess, or perforation”) were observed without surgery or antibiotic therapy. Their series showed a long-term event free rate of 60%, with recurrences at an average of two years post-diagnosis 19.

In a multi-center study, Bachur et al. found that the use of ultrasound in boys less than 5 years-old increased negative appendectomy rates 20. Bachur et al.’s study relied on coding data, and therefore could not arrive as a mechanism that would explain this increase in negative appendectomy rate. Perhaps appendicitis was overcalled by an overreliance on secondary signs, and perhaps the appendicitis resolved by the time of surgery.

In children, 21% of radiology-performed ultrasounds contain language that renders diagnosis of appendicitis uncertain 21. Our cases may have represented appendiceal thickening as the sonographic sign of enteritis. Early appendicitis without fecalith may be overcome by a healthy immune system, like other bacterial infections. Complicated appendicitis is unusual before 12 hours of abdominal pain 22, and repeating sonography after a brief observation may eliminate the burden of cases that have resolved. Others demonstrate that pediatric perforated appendicitis occurs more commonly after the first 24 hours of symptoms 23, 24. In Narsule’s study of 197 children with appendicitis, none with less than 12 hours of symptoms were perforated at time of surgery 25. Thus, observation in cases of very early appendicitis may be reasonable.

Children may develop chronic appendicitis, characterized by intermittent, colicky right lower quadrant pain and fibrosis of the appendix on pathological examination 26. Chronic appendicitis is thought to be related to a partial or chronic appendiceal obstruction, and the incidence in children is unknown. We suggest that spontaneously resolving appendicitis may be an episode within the trajectory of chronic appendicitis.

### Limitations

Our study is retrospective and did not provide a CT or pathologic confirmation of appendicitis. As these cases review sonographic appendicitis without a confirmatory study, we cannot exclude that the appendix was inflamed in the context of a developing gastrointestinal illness that subsequently resolved. Our PED does not use a validated scoring system to assess the pretest probability of appendicitis. Our PED patients are examined by physicians at different levels of training, and we therefore felt that the physical examination documentation was subjective and not sufficiently reliable to create an appendicitis pretest score retrospectively. 

## Conclusion

Children with POCUS findings suggestive of appendicitis may not necessarily need treatment (antibiotics or surgery) prior to a certain duration of symptoms as symptoms may self-resolve. Cases that present with only several hours of abdominal pain may warrant a brief observation and repeat ultrasound if the clinical context results a low pretest probability for clinically significant appendicitis. Patients who experience spontaneous resolution of appendicitis may be at an increased risk of recurrence.

## Patient Consent

Patient consent for a case series is not required by our institutional review board.

## Conflict of Interest Statement

 The authors declare no relevant conflicts of interest.

## Financial Support

 We declare no financial support/sponsorship.

## Supplementary Material

 Video S1 7-millimeter appendix with wall thickening in long-axis.
